# The Association between Autism Spectrum Disorder and Pre- and Postnatal Antibiotic Exposure in Childhood—A Systematic Review with Meta-Analysis

**DOI:** 10.3390/ijerph16204042

**Published:** 2019-10-22

**Authors:** Eunmi Lee, Jeonghyun Cho, Ka Young Kim

**Affiliations:** 1 Department of Nursing, Research Institute for Basic Sciences, Hoseo University, Asan 31499, Korea; sweetbear2@hanmail.net; 2 Department of Nursing, College of Medicine, Inje University, Busan 47392, Korea; jhcho@inje.ac.kr; 3 Department of Nursing, College of Nursing, Gachon University, Incheon 21936, Korea

**Keywords:** antibiotic exposure, autism spectrum disorder (ASD), childhood, meta-analysis, systematic review

## Abstract

Autism spectrum disorder (ASD) is a developmental disorder that begins in early childhood and has been associated with several environmental and genetic factors. We aimed to conduct two-side meta-analyses to determine the association between ASD and pre- and postnatal antibiotic exposure in childhood. We searched PubMed, Embase, Web of Science, and Cochrane Library for articles published up to February 2019. We evaluated observational studies that assessed the association between ASD and antibiotic exposure. Of 1459 articles, nine studies were used in the meta-analysis. We found that early antibiotic exposure, including pre- and postnatal, significantly increased the ASD risk in children. Furthermore, early antibiotic exposure, including pre- and postnatal, was significantly increased in children with ASD. Specifically, prenatal antibiotic exposure was significantly increased in children with ASD; however, postnatal antibiotic exposure was not. Our results indicate an association between ASD and early antibiotic exposure; specifically, that prenatal antibiotic exposure is an important risk factor of ASD in children.

## 1. Introduction

Autism spectrum disorder (ASD) is a neurodevelopmental disorder characterized by communication and interaction difficulties as well as repeated and stereotyped behaviors and interests [1]. Approximately 66 million individuals worldwide have ASD, and it has been reported to begin early in life [2,3,4]. 

Although the exact cause of ASD is unknown, genetic factors are key contributors. Further, various environmental factors are involved and are considered an important aspect of the increased prevalence of ASD [5,6,7]. Rapid industrialization and the consequent environmental pollution have also increased the risk of exposure to toxic substances via various pathways. Since ASD occurs early in life, studies have reported that toxic exposure, such as mercury poisoning in the prenatal or childhood period, could cause ASD [8]. Therefore, infant mercury poisoning caused by seafood consumption by the mother, pediatric vaccination, and prenatal use of antibiotics has been suggested to cause ASD [9]. Moreover, studies have reported that pre- [10,11,12] and postnatal exposure to antibiotics and acetaminophen increases the ASD risk [13,14,15,16]. 

Although antibiotics are essential for treating some diseases, antibiotic exposure has been associated with various diseases. Antibiotic use can cause abnormal changes in the human microbiome. Studies have suggested that when the altered microbiome is passed onto the fetus, it increases the risk of colitis and ASD by impeding neurological development [13,17,18,19]. Further, antibiotic exposure has been reported to be weakly associated with the risk for ASD [10]; however, this association cannot be clearly established given the difficulty of measuring fetal antibiotic exposure. Studies have also reported that postnatal antibiotic exposure is associated with ASD; specifically, postnatal use of acetaminophen and antibiotics, as well as ear infection, were reported to increase the risk for ASD [16]. 

This indicates that despite continued studies on the association between ASD and antibiotics, a definitive conclusion is yet to be reached. Particularly, the lack of consensus on pre- and postnatal antibiotic exposure has hindered efforts toward determining this relationship. Therefore, we aimed to conduct two-side meta-analyses to clearly determine the relationship between ASD and antibiotic use as well as present foundational data for preventive and management strategies for lowering the ASD risk.

## 2. Methods

### 2.1. Search Strategy and Selection Criteria

This meta-analysis was performed in accordance with the PRISMA guidelines [20] and was approved by the institutional review board of Gachon University (1044396-201909-HR-160-01). We searched PubMed, Embase, Web of Science, and Cochrane Library databases for all relevant studies written in English and published before February 2019. The search terms used were (antibiotic* OR antimicrob*) and (autism* OR ASD OR kanner syndrome OR PDD OR pervasive developmental disorder). For meta-analysis, we included studies that reported the association between ASD and pre- or postnatal antibiotic exposure in childhood and excluded studies that did not report the antibiotic exposure level as well as the ASD and control group. We included observational studies, including case-control, cross-sectional, and cohort studies and excluded commentary, editorial, and review articles. 

### 2.2. Data Extraction and Analysis

All authors independently screened and selected relevant data, and any disagreements were resolved by discussion. We extracted relevant data, including first author, publication year, study design, country, sample size, age of participants, and related outcomes. Moreover, to assess the association between ASD and pre- or postnatal antibiotic exposure in childhood, we extracted all measurement data including prevalence data of patients with ASD and controls group as well as the number and amount of pre- and postnatal antibiotic exposure. The risk estimates of ASD in antibiotic exposure and the standardized mean difference (SMD) of antibiotic exposure in ASD were analyzed via meta-analysis using the Comprehensive Meta-Analysis software version 3 (Biostat Inc., Englewood, NJ, USA). We assessed the heterogeneity using the Q statistic and *I*^2^ method, while the random-effects model was applied in all analyses to address heterogeneity. Subgroup analyses were performed based on group differences in prenatal and postnatal exposure. Publication bias was assessed using funnel plots and Egger’s intercept. *p* < 0.05 indicated a significant difference. Furthermore, for quality assessment, we used the Newcastle–Ottawa Scale (NOS) to assess the methodological quality of the nine included studies [21,22]. The NOS is a representative tool developed for meta-analysis of non-randomized comparative studies, including case-control, cross-sectional, and cohort studies. It comprises an adjustment item with the selection of study groups, comparability of the groups, and determination of either the exposure or outcome of interest to evaluate the quality of articles included in the meta-analyses.

## 3. Results

### 3.1. Characteristics of the Studies

**Figure 1:**
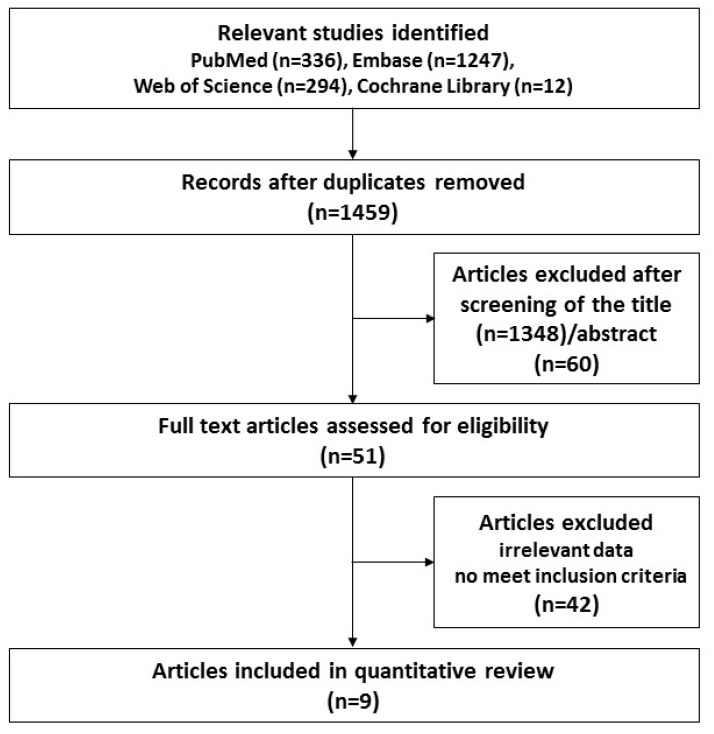
Flow chart for the study selection process.

We initially identified 1877 studies (Figure 1), and after the elimination of duplicates using the Endnote reference database, 1459 studies remained. We excluded 1348 articles based on the titles and 60 articles based on the abstract, and thus were left with 51 articles that were eligible for full-text screening. After close review, 42 publications were excluded because of lack of usable data and failure to meet the inclusion criteria. Finally, a total of nine articles were identified and included in the meta-analysis. Table 1 shows the characteristics of the nine selected studies, which were published between 2007 and 2019. Moreover, regarding study design, there were 5, 1, and 3 case-control, cross-sectional, and cohort studies, respectively. The subjects ranged from 27 to 949,821. All studies were performed in the USA, Denmark, Italy, Lebanon, Canada, and Sweden. The age of the subjects ranged from birth to 18 years. The outcomes of the nine studies were only described as related content regarding ASD and antibiotic usage. In addition, the quality scores ranged from 5 to 8.

### 3.2. Meta-Analysis Result Relating the ASD Risk in Antibiotic Exposure

**Figure 2:**
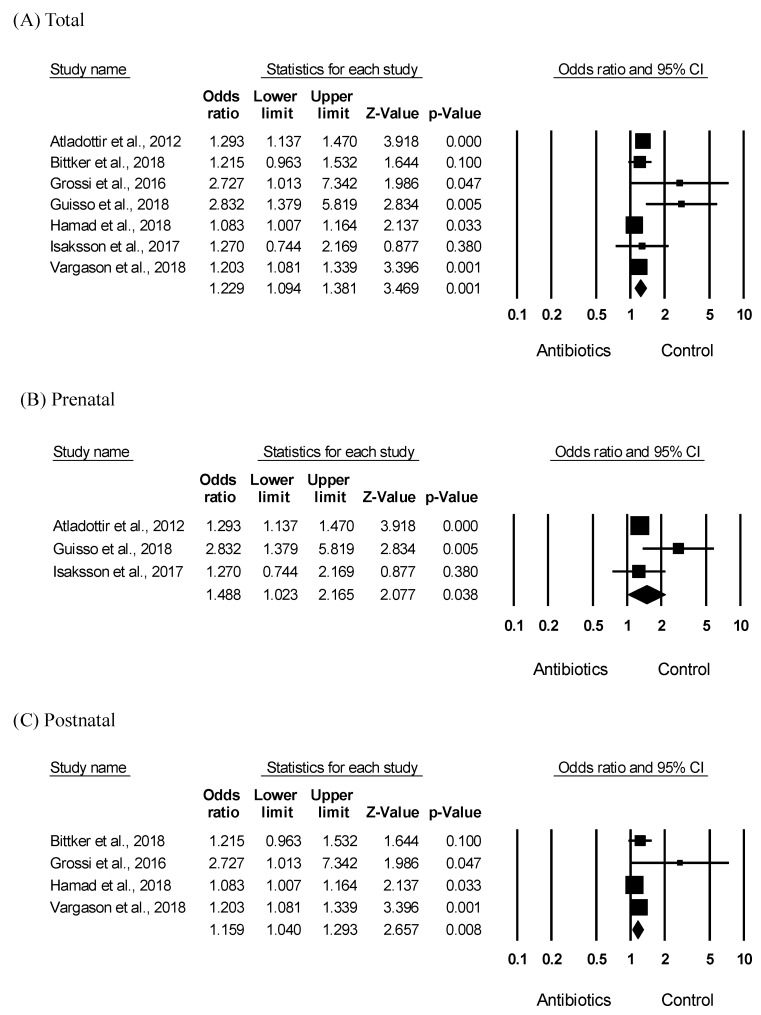
Meta-analysis of the odds ratio estimate of ASD risk according to antibiotic exposure in children. Effects sizes are measured as odds ratio in the antibiotic exposed group compared to that in the control group. (**A**) Forest plot of the ASD risk in early (pre- and postnatal) antibiotic exposure. Heterogeneity, Q = 15.669, *p* = 0.016, *I*^2^ = 61.709 (**B**) Forest plot of the ASD risk in prenatal antibiotic exposure. Heterogeneity, Q = 4.441, *p* = 0.109, *I*^2^ = 54.966 (**C**) Forest plot of the ASD risk in postnatal antibiotic exposure. Heterogeneity, Q = 6.075, *p* = 0.108, *I*^2^ = 50.619.

To investigate the association between ASD and antibiotic exposure, the ASD risk was analyzed based on the antibiotic exposure and control group. As shown in Figure 2A, meta-analysis results indicated that early antibiotic exposure, including pre- and postnatal, significantly increased the ASD risk in children (OR = 1.229, 95% CI: 1.094–1.381, *p* = 0.001) with moderate heterogeneity (*I*^2^ = 61.71). As shown in Figure 2B, the results of subgroup analyses showed that prenatal antibiotic exposure significantly increased the ASD risk (OR = 1.488, 95% CI: 1.023–2.165, *p* = 0. 038) with moderate heterogeneity (*I*^2^ = 54.97). Furthermore, as shown in Figure 2C, postnatal antibiotic exposure significantly increased the ASD risk in children (OR = 1.159, 95% CI: 1.040–1.293, *p* = 0.008, *I*^2^ = 50.62). Moderate heterogeneity was identified across all studies; therefore, as shown in Figure 2, a random-effects model was performed. The funnel plots and Egger’s test (*p* = 0.03) showed significant results in seven of the included studies; however, in subgroup analyses, the results of the Egger’ tests (Figure 2B; *p* = 0.50, Figure 2C; *p* = 0.13) were not significant, indicating no publication bias. 

### 3.3. Meta-Analysis Results Related to Antibiotic Exposure in ASD

**Figure 3:**
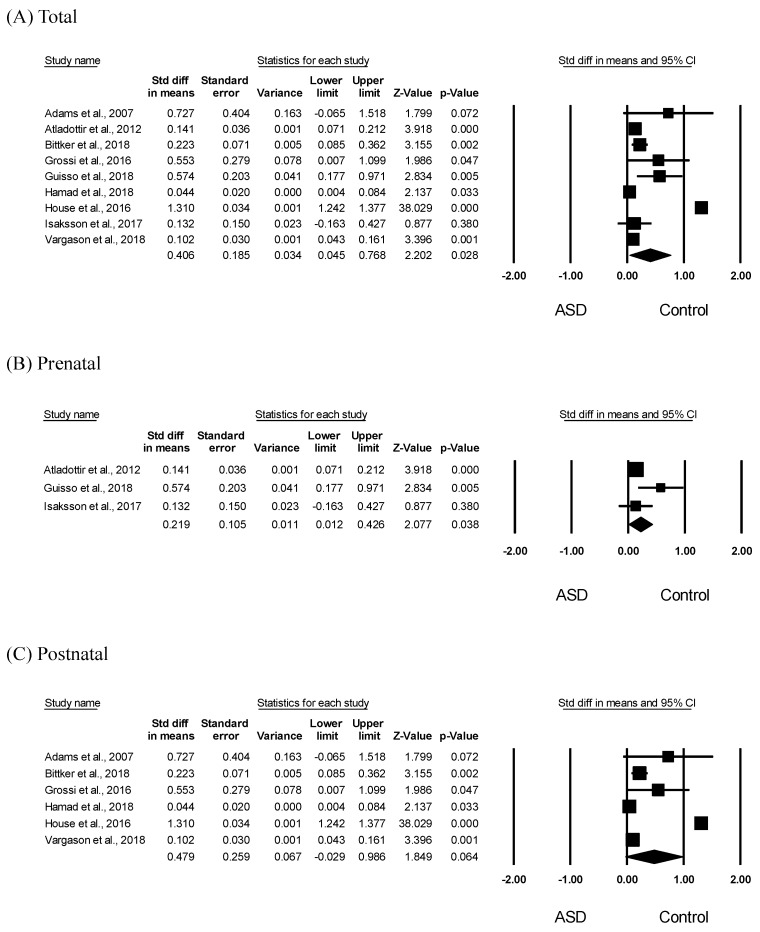
Meta-analysis of early antibiotic exposure in children with ASD. Effects sizes were measured as standardized mean difference in the ASD group compared to that in the control group. (**A**) Forest plot of early (pre- and postnatal) antibiotic exposure in children with ASD. Heterogeneity, Q = 1082.915, *p* < 0.001, *I*^2^ = 99.261 (**B**) Forest plot of prenatal antibiotic exposure in children with ASD. Heterogeneity, Q = 4.441, *p* = 0.109, *I*^2^ = 54.966 (**C**) Forest plot of postnatal antibiotic exposure in children with ASD. Heterogeneity, Q = 1062.851, *p* < 0.001, *I*^2^ = 99.530.

To assess the association between ASD and antibiotic exposure in children, we analyzed differences in antibiotic exposure between the ASD and control groups. Meta-analysis results showed significantly increased early antibiotic exposure, including pre- and postnatal, in children with ASD (SMD = 0.406, 95% CI: 0.045–0.768, *p* = 0.028) (Figure 3A). Further, there was obvious heterogeneity in the included studies (*I*^2^ = 99.26); therefore, we applied a random-effects model to the analysis. The funnel plots and Egger’s test did not give significant results (*p* = 0.67), indicating no publication bias. In the subgroup analyses, prenatal antibiotic exposure was significantly increased in children with ASD (SMD = 0.219, 95% CI: 0.012–0.426, *p* = 0.038) with moderate heterogeneity (*I*^2^ = 54.97) (Figure 2B). However, as shown in Figure 3C, there was no significant difference in the postnatal antibiotic exposure between children with ASD and controls (SMD = 0.479, 95% CI: −0.029 to 0.986, *p* = 0.064, *I*^2^ = 99.53). As shown in Figure 3, there was significant heterogeneity across all studies; therefore, a random-effects model was performed. Furthermore, the funnel plots and Egger’s test performed for all studies did not show statistical significance (Figure 3A; *p* = 0.67. Figure 3B; *p* = 0.50, Figure 3C; *p* = 0.67), indicating no publication bias.

## 4. Discussion

This study analyzed the relationship between ASD and early antibiotic use in children using two-sided meta-analyses. Specifically, we analyzed the relationship between ASD and pre- and postnatal antibiotic exposure in children using a systematic method. Our results indicated that pre- and postnatal antibiotic use increased the ASD risk. This is consistent with previous reports that pre- and postnatal antibiotic use causes ASD in children [10,13,17,18,19]. In our study, both pre- and postnatal antibiotic use increased the ASD risk (Figure 2); however, only prenatal, but not postnatal, antibiotic use was significantly elevated in children with ASD compared to controls (Figure 3). It is difficult to compare or discuss the risk levels of prenatal and postnatal antibiotic use because studies on the relationship between ASD risk and postnatal antibiotic use did not specify the prenatal antibiotic exposure [16,24]. Further, studies on the relationship between ASD onset and prenatal antibiotic use did not specifically mention a follow-up on postnatal antibiotic exposure [12]. However, the results of our bidirectional meta-analysis suggest that prenatal antibiotic exposure is a more potent risk factor for ASD. This is further indicated by a pre-birth cohort study that examined the association between pre- and postnatal smoking exposure and atopic eczema, and reported that only prenatal smoking exposure increased the risk of atopic eczema by 7.11 times compared to postnatal smoking exposure [29]. Moreover, ASD, which is an early complex neurodevelopmental disorder, is known to develop before the age of three [30]. In particular, the fetal period is a crucial period in brain development and exposure to harmful environmental factors during this period can substantially increase the risk of neurodevelopmental disorders [31]. One study reported that prenatal opiate exposure affected brain development [32]. Therefore, prenatal antibiotic exposure is believed to be an important risk factor for ASD onset [33,34]. Indeed, early postnatal antibiotic exposure is known to increase ASD risk. However, in our study, the reduced influence of postnatal antibiotic exposure on increasing ASD risk could be attributed to the age range of our study population (birth to 18 years). Further studies comparing pre- and postnatal antibiotic use with adjustment for various confounders should be conducted to confirm our results.

Although we systematically analyzed the relationship between ASD and early antibiotic exposure, this study has a few limitations. First, although we analyzed various data on antibiotic exposure, including the number and amount of exposure as well as diverse antibiotic mechanisms, we could not analyze according to antibiotic type due to insufficient data. Further, we did not consider the gestational age in prenatal exposure and used a wide age range for postnatal exposure. Second, although we used a random-effects model to address the high heterogeneity and indicated no publication bias, except for the total results relating to ASD in early exposure, we had a limited sample size. Third, postnatal antibiotic exposure remains controversial. Although the test results on publication bias showed low statistical significance, they cannot be considered conclusive on their own given the small sample size and their closeness to statistical significance. There has been ambiguity in distinguishing different periods in previous studies on the effects of pre- and postnatal antibiotic use. Further, small experimental and large investigative studies reported contradicting findings due to the difficulty of adjusting for various confounders, which were described as limitations. Thus, there is a need for large controlled experimental or cohort studies adjusting for potential confounders to clearly determine the impact of postnatal antibiotic exposure on ASD. 

## 5. Conclusions

Notwithstanding these limitations, this study remains significant since it used two-sided meta-analyses to systematically analyze the relationship between ASD and early antibiotic exposure and provides more evidence on the association between ASD risk and prenatal antibiotic exposure. Subsequent studies should further analyze the association of pre- and postnatal antibiotic exposure with ASD to contribute towards lowering the ASD risk and the prevention and management of ASD in children. 

## Figures and Tables

**Table 1:** Characteristics of the 9 included studies on the association between antibiotic exposure and autism spectrum disorder (ASD) in children.

Study	Study Type	Participants	Location	Age	Outcome	Quality *
Adams et al., 2007 [15]	Case-control	27	USA	3–8 years	Children with autism showed higher oral antibiotic usage in various time periods.	5
Atladóttir et al., 2012 [12]	Cohort	976	Denmark	8–14 years	The use of various antibiotics showed an increased risk of ASD/infantile autism.	7
Bittker et al., 2018 [16]	Case-control	1515	USA	3–12 years	Postnatal antibiotic use was associated with an increased ASD risk.	6
Grossi et al., 2016 [23]	Case-control	113	Italy	5–16 years	Regarding potential risk factors, the frequency of early antibiotic use was higher in the autism group than in the typical group.	7
Guisso et al., 2018 [24]	Case-control	314	Lebanon	2–18 years	In the multivariable analysis, antibiotics were negatively associated with ASD.	7
Hamad et al., 2018 [25]	Cohort	214,834	Canada	2–8 years	Children who received antibiotics had a reduced ASD risk.	7
House et al., 2016 [26]	Cross-sectional	949,821	USA	0–17 years	The rate of antibiotic use among children diagnosed with ASD was more than 2-fold that of the general population.	8
Isaksson et al., 2017 [27]	Case-control	415	Sweden	4–9 years	An environmental risk index based on the use of antibiotics during pregnancy and/or the breastfeeding period was associated with ASD.	7
Vargason et al., 2019 [28]	Cohort	281,623	USA	0–5 years	Greater numbers of oral antibiotic fills during the first 3 years of enrollment were found to significantly increase the hazard rate of having a later GI-related diagnosis in both children with and without ASD.	7

* Quality was assessed using the Newcastle–Ottawa Scale.
